# Acinar Atrophy, Fibrosis and Fatty Changes Are Significantly More Common than Sjogren’s Syndrome in Minor Salivary Gland Biopsies

**DOI:** 10.3390/medicina58020175

**Published:** 2022-01-24

**Authors:** Ainat Klein, Jonathan Klein, Moran Chacham, Shlomi Kleinman, Amir Shuster, Oren Peleg, Clariel Ianculovici, Ilana Kaplan

**Affiliations:** 1 Neuro-Ophthalmology Unit, Ophthalmology Division, Tel-Aviv Sourasky Medical Center, Sackler Faculty of Medicine, Tel-Aviv University, Tel-Aviv 6997801, Israel; kleinainat@gmail.com; 2 4 Lea Imenu St, Modiin 7176253, Israel; Jonathankln@yahoo.com; 3 11 Tiber St, Givataim 5341506, Israel; moranchacham1@gmail.com; 4 Department of Otolaryngology, Head and Neck Surgery and Maxillofacial Surgery, Tel-Aviv Sourasky Medical Center, Tel-Aviv 6423906, Israel; dr.shlomi@gmail.com (S.K.); shusterim@gmail.com (A.S.); orenpeleg@gmail.com (O.P.); dr.clariel@gmail.com (C.I.); 5 Department of Oral Surgery, Goldschleger School of Dental Medicine, Tel-Aviv University, Tel-Aviv 6997801, Israel; 6 Department of Oral Pathology, Oral Medicine and Maxillofacial Imaging, Goldschleger School of Dental Medicine, Tel-Aviv University, Tel-Aviv 6997801, Israel

**Keywords:** Sjögren, IgG4, salivary glands, xerostomia, xerophthalmia

## Abstract

*Background and Objective:* Hyposalivation and xerostomia can result from a variety of conditions. Diagnosis is based on a combination of medical history, clinical and serological parameters, imaging, and minor salivary gland biopsy when indicated. The Objective was to characterize microscopic changes in minor salivary gland biopsies taken in patients with xerostomia. *Materials and Methods:* 10-year retrospective analysis of minor salivary gland biopsies, 2007–2017. Histomorphometric analysis included gland architecture, fibrosis, fat replacement, inflammation and stains for IgG/IgG4, when relevant. *Results:* 64 consecutive biopsies, of which 54 had sufficient tissue for diagnosis of Sjogren’s Syndrome (SS) were included (18 males, 46 females, average age 56 (±12.5) years). Only 12 (22.2%) were microscopically consistent with SS, none stained for IgG4. Medical conditions were recorded in 40 (63%), most frequently hypertension and hyperlipidemia (28% each). Medications were used by 45 (70%), of which in 50% more than one. Xerostomia in non-SS cases was supported by abnormal gland morphology, including acinar atrophy, fibrosis and fatty replacement. All morphological abnormalities are correlated with age, while fatty replacement correlated with abnormal lipid metabolism. Multiple medications correlated with microscopic features which did not correspond with SS. *Conclusions:* SS was confirmed in a minority of cases, while in the majority fatty replacement, fibrosis and multiple medications can explain xerostomia, and are related to aging and medical conditions. Medical history and auxiliary tests could lead to correct diagnosis in non-SS patients, avoiding biopsy. The necessity of a diagnostic biopsy should be given serious consideration only after all other diagnostic modalities have been employed.

## 1. Introduction

Saliva is secreted into the oral cavity from the major and minor salivary glands. A normal physiological production of saliva is 0.5–1.5 L per day. Decreased saliva production can cause functional impairment such as changes in taste, halitosis, difficulty in chewing, swallowing and speech. It also increases the risk of caries and causes dental sensitivity. The causes for decreased production include old age, side effects of many types of medications, chemotherapy and radiation to the head and neck area. Some autoimmune conditions such as Sjogren’s syndrome (SS) and IgG4 related disease (IgG4RD) and neurological diseases have also been associated with hyposalivation [1].

Xerostomia, the subjective feeling of dry mouth, is common with an increasing incidence mainly attributed to increased life expectancy [1].

SS is a systemic chronic autoimmune disease that causes inflammation and dysfunction of the salivary glands and may lead to severe decrease in the lacrimal and salivary secretions. It may be primary, if it is limited to the execratory glands, or secondary, when accompanied with at least one additional inflammatory condition [1,2]. Diagnosis of SS is based on a combination of objective and subjective signs of which at least four are mandatory for a diagnosis of SS, and two must be objective parameters. These include symptoms of dry mouth, symptoms of dry eyes, Schirmer test proving xerophthalmia, sialometry below the threshold for hypofunction, sialography and biopsy of minor salivary glands of the lower lip [3,4]. A biopsy is only one of the objective criteria that can be used for diagnosis of SS [3,4].

IgG4-RD is a multi-organ immune-mediated fibro-inflammatory condition, with a core of pathological, clinical and serologic similarities [5,6,7,8,9] The common findings include tumor-like swelling of involved organs, a lymphoplasmacytic infiltrate enriched in IgG4-positive plasma cells, and a variable degree of fibrosis, with a characteristic storiform pattern. Up to 60–70% of patients exhibit increased levels of serum IgG4 [7,8,9,10]. Major salivary gland involvement is a common feature of IgG4-RD. Since there is not much literature on minor salivary gland involvement, the various manifestations of IgG4-RD and its relation to sialadenitis has only been recently described, older pathological samples were not routinely tested for its presence.

This study aimed to histomorphometrically investigate microscopic changes in the minor salivary glands in patients with xerostomia, search for correlations to systemic diseases, medications used and serum markers. In addition, a re-evaluation for IgG4RD markers in minor salivary gland pathology specimens was performed.

## 2. Materials & Methods

The study was a retrospective analysis of archival minor salivary gland biopsies obtained between January 2007 and December 2017. The inclusion criteria were biopsies obtained from patients with xerostomia suspected of having SS. Only adult patients over 18 years were included. Exclusion criteria were a previous history of major surgery or radiation to the head and neck area.

The demographic and clinical parameters collected from patient’s files included age, gender, previous medical diagnosis, medications, chief complaint, reason for biopsy referral, the referring physician specialty, and previous treatment. Additional laboratory parameters collected from the medical files included serum markers Rheomatoid Factor (RF), Anti-nuclear Antibody (ANA), IgG4, Sjogren Syndrome antibody A (SSA) and Sjogren’s syndrome antibody B (SSB). 

The pathological slides were retrieved from the medical archive and were re-evaluated for the following parameters: the number of lobules of salivary gland tissue present (at least five required for diagnosis of SS); preservation of normal gland-architecture (lobules, secretory ducts, acini) were evaluated semi-quantitatively on a three-tier scale (well preserved, partially preserved, not preserved); fibrosis involving the gland parenchyma (three-tier scale; 1 = up to 25% of gland surface, 2 = up to 50%, 3 = above 50%); replacement of gland tissue by adipose tissue (fatty replacement) relative to gland area; lymphocyte infiltration was classified as focal or diffuse. In focal infiltrates the density was calculated, according to criteria for SS [11]. The presence of plasma cells was recorded in a binary fashion, an Ig/IgG4 ratio, in relevant cases [12].

Statistical analysis: a Mann-Whitney test was performed to compare quantitative variables. For comparing categorical variables, a Chi-square test was performed. In cases of a minority of subjects in the comparison groups, the Pearson correlation coefficient test was performed.

## 3. Results

During the study period, 69 pathology specimens fulfilling the inclusion criteria have been retrieved. Four cases were excluded due to absent clinical data in the hospital medical records, one patient had two biopsies performed during this time period, but only one was included, with a total of 64 samples included in the statistical analysis. 

The cohort included 18 males (28%) and 46 females (72%), with an average age of 56 years (+/−12.5). For the correlation analysis between clinical parameters and biopsy results for SS, only 54 cases were included, since 10 samples did not contain the minimum number of salivary gland lobules to be acceptable for diagnosis of SS [13]. For correlation analysis between the pathological features (fibrosis, fat changes, inflammatory infiltration and clinical parameters) all samples (n = 64) were included. 

The main reason for biopsy referral was xerostomia (91%), of which 35 (60%) also recorded symptoms of xerophthalmia. 

Forty (62.5%) patients referred for salivary gland biopsy had pre-existing medical conditions, most frequently hyperlipidemia 18 (28%) and hypertension 18 (28%). Other medical conditions recorded included gastro esophageal reflux disease (GERD) 11 (17%), fibromyalgia 4 (6%), arthritis 5 (8%), and other autoimmune diseases 5 (8%). Correlations between microscopic features of SS and multiple parameters were investigated, including a spectrum of medical conditions, patient complaints, smoking, results of Schirmer test, serology for SSA, SSB yielded significant correlation only between positive serology for SSA or SSB and microscopy consistent with SS (*p* = 0.004, *p* = 0.02, respectively) (Table 1).

Systemic medications were recorded in 45 (70%), of which almost 50% used more than one prescribed medication. 

Table 2 presents the analysis of the cohort of 54 patients, in which the sample was accepted for pathological diagnosis/exclusion of SS. There were 14 males (26%) and 40 females (74%), with an average age of 56 years.

In 12 (22.2%) of the pathological findings were consistent with SS. There were no statistically significant differences in age or gender between this subgroup and the remaining cases. There was no correlation between other systemic disease nor any specific medication in use with a biopsy result consistent with SS.

**Figure 1:**
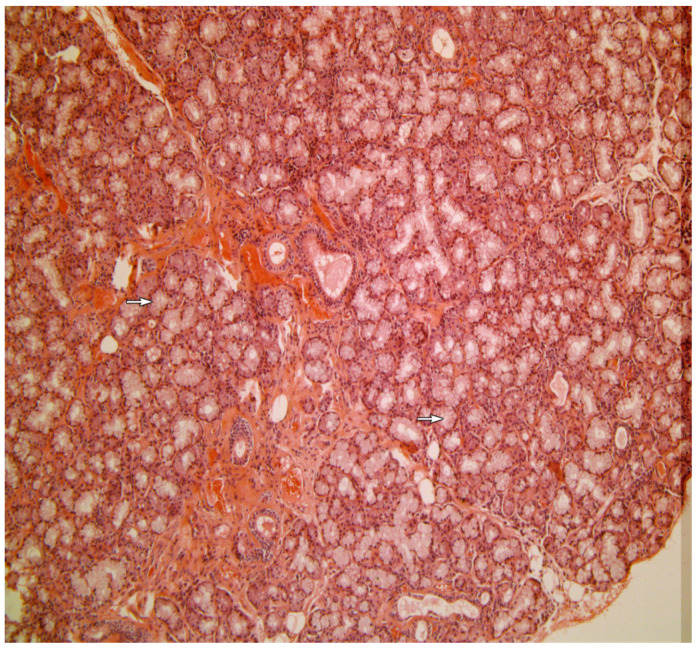
A normal preserved minor salivary gland with no significant pathological changes (Hematoxylin and Eosin, original magnification ×40). The arrows point to normal acini.

**Figure 2:**
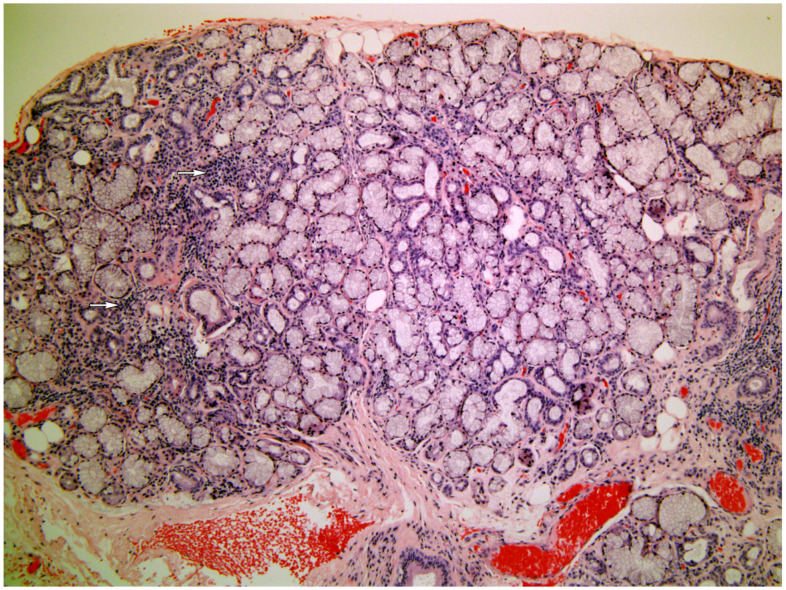
The normal gland structure is preserved, with focal inflammatory.infiltrate (arrows), typical for Sjögren syndrome(Hematoxylin and Eosin, original magnification ×40).

**Figure 3:**
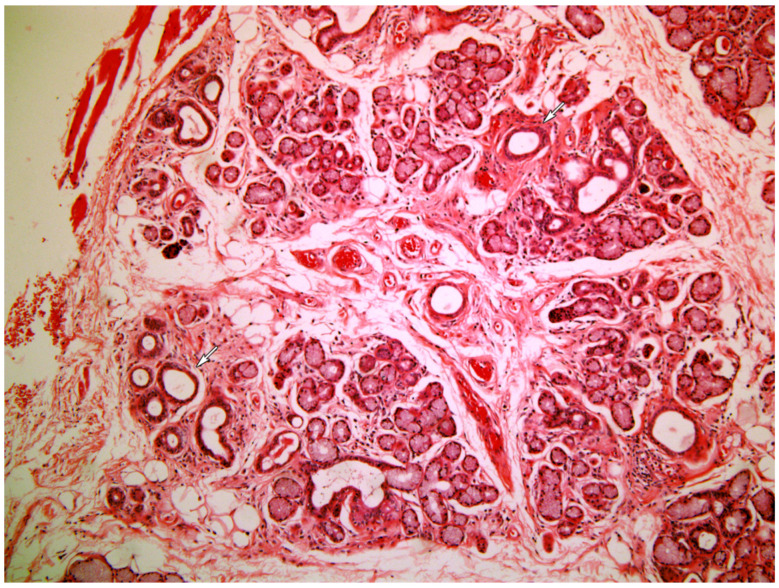
Salivary gland displaying focal atrophic changes of the acini (arrows). (Hematoxylin and Eosin, original magnification ×40).

**Figure 4:**
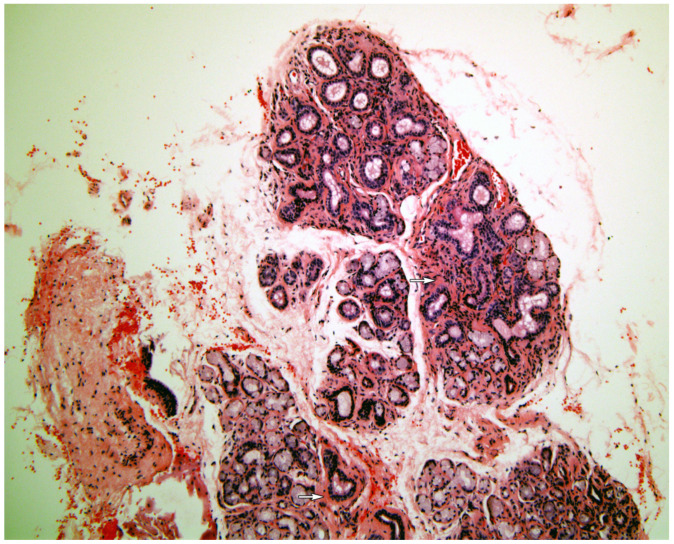
A minor salivary gland with severe acinar atrophy and marked fibrosis. (arrows). There is very little inflammation visible. (Hematoxylin and Eosin, original magnification ×40).

**Figure 5:**
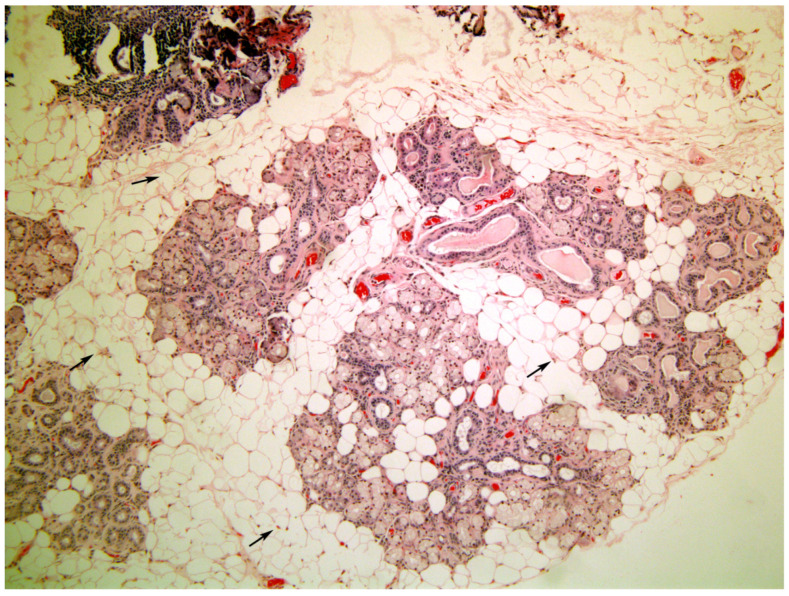
Marked fatty changes (arrows), replacing most of the glandular parenchyma. (Hematoxylin and Eosin, original magnification ×40).

Figure 1, Figure 2, Figure 3, Figure 4 and Figure 5 present typical findings of normal morphology (Figure 1), SS (Figure 2), focal acinar atrophy (Figure 3), severe atrophy and fibrosis (Figure 4) and fatty replacement (Figure 5).

A biopsy consistent with SS correlated with fewer medications in use, and a negative correlation was found between the use of more than three medications and a biopsy consistent with SS (*p* = 0.05). 

Only six patients had recorded results of Schirmer test (ST), of which one had an ST consistent with SS but a negative biopsy, and five tests were inconsistent with SS (three biopsy results inconsistent with SS and two biopsy results consistent with SS). The groups’ size was too small for statistical analyses. 

Regarding the field of specialty of the referring physician, 39% of patients were referred by a physician from the oral and/or dental health (oral pathology expert, oral and maxillofacial surgeon), the remaining 61% were referred by rheumatologist, ear nose and throat or internal medicine experts. Of the biopsies referred by an oral expert, 50% were found to be consistent with SS, while in only 19% of the other cases referred by medical physicians the biopsies supported the diagnosis of SS. These differences did not reach the threshold for statistical significance. 

Among patients with biopsy results supporting SS, there were more patients positive for SSA, SSB (*p* = 0.02, 0.004) than in patients with microscopic features inconsistent with SS. A positive biopsy for SS was obtained in 66.6% of patients expressing SSB and 71.4% of those expressing SSA. 

There were only four specimens that were suspicious of IgG4RD, with fibrosis grade 2 and above (more than 25% of the area), and with the presence of plasma cells. However, when immunostains for IgG4 were performed, none was found to be positive to IgG4. 

The histomorphometric analysis was initially performed in a semi-quantitative method, evaluating fibrosis and fatty changes on a four-tier scale. However, this sub categorization led to too small numbers of cases in each group; thus, dichotomy re-grouping was performed (with and without fibrosis, with or without fat infiltration (see Table 2)). A strong correlation was found between age and fatty replacement (*p* = 0.01), age and fibrosis (*p* = 0.023), as well as between age and acinar atrophy (*p* = 0.003). 

Fibrosis was more prevalent among patients negative for SSB (*p* = 0.039). 

There was positive correlation between fatty replacement and hyperlipidemia (*p* = 0.004) and between fatty replacement and multiple medication in use (*p* = 0.024). Acinar atrophy was more prevalent among females: 81.8% of samples with atrophy belong to females, while among patients with no atrophy there were 57.7% female. This was statically significant (*p* = 0.043). 

## 4. Discussion

Symptoms of dry mouth (xerostomia) are common, especially among older patients. These symptoms have been shown to correlate with objective salivary hypofunction, by different collection methodologies [13].

The most prevalent reason for salivary hypofunction is medication use, as several hundred medications have a recognized xerostomic effect, including many commonly prescribed medications such as cardiovascular medications, mood altering compounds, medications for gastrointestinal conditions, lipid metabolism medications and many other types [1,14,15]. This is even more common in patients using polypharmacy, defined as three or more medications. Dry eyes are also a common finding in these patients [16]. However, for diagnosis of drug-related salivary hypofunction, as well as age-related hypofunction, strictly defined criteria have not been established.

Other rare congenital conditions which may impair saliva production include structural anomalies of the parenchyma or ductal system, such as aplasia and artresia [17]. However, these are recognized in childhood, whereas most patients in which a work-up for xerostomia is performed are adults and the elderly.

The present results have been able to show that in cases in which SS was ruled out, the biopsies did not show normal gland morphology. On the contrary, a spectrum of abnormal morphological changes was demonstrated, including acinar atrophy, fibrosis and fatty replacement of the parenchyma: all of which can explain the symptom of xerostomia, as they reflect a significant decrease in the gland component responsible for the production of saliva. Variable amounts of inflammation were present as well, but the pattern was distinctively different than in SS, with a diffuse distribution rather than the focal pattern which is diagnostic for SS.

These morphological changes correlated with increased age and with polypharmacy, a well-known connection between aging and medications used. The present cohort confirms that polypharmacy can be associated with microscopic changes in the glands, such as fibrosis and inflammation, but that the pattern is different than the findings diagnostic for SS. Similar results have been reported before [14,15].

SS is a systemic, chronic autoimmune disease that causes inflammation and dysfunction of the salivary glands and may lead to severe decreases in lacrimal and salivary secretions. It usually affects women between 30 and 60 years of age. The syndrome is considered primary when it is limited to the execratory glands, and secondary when it is accompanied by at least one additional inflammatory disease (rheumatoid arthritis, scleroderma, lupus erythematosus and other connective tissue diseases) [1,2]. Over the years, various sets of diagnostic criteria have been suggested, combining clinical features, serology and biopsy of minor salivary glands from lower lip. According to the recent American-European consensus paper, a set of six objective and subjective parameters has been suggested, of which at least four are mandatory for a diagnosis of SS, and two must be objective parameters. These include symptoms of dry mouth, symptoms of dry eyes, a Schirmer test proving xerophthalmia, sialometry below the threshold for hypofunction, sialography and biopsy of minor salivary glands of the lower lip [4]. A biopsy is thus optional but not mandatory if sufficient other criteria are met. If a biopsy is obtained, diagnostic criteria include focal lymphocytic sialadenitis (a focus is at least 50 lymphocytes) and the mean density of lymphocytic foci calculated must be ≥1 focus per 4 mm^2^ (focus score ≥ 1). The biopsy should also contain at least five lobules of salivary gland tissue to be considered acceptable for diagnosis [3,4,11]. SS cases may exhibit acinar atrophy and replacement by the inflammatory infiltrate, but they usually do not have significant fibrosis or fatty replacement.

The present retrospective study group, in which only 22.2% received a final diagnosis of SS, raises questions on the necessity of a biopsy for the diagnosis in the majority of patients referred for biopsy during work-up for xerostomia. In older patients, with systemic conditions such as high blood pressure and/or hyperlipidemia, which usually also use multiple medications, sufficient clinical information may be present to explain the complaint of xerostomia, without the need for a biopsy. Serology negative for markers of SS can also help in rejecting the possibility of SS, while dry eyes are not conclusive, because the same medications inducing xerostomia are also known to induce xerophthalmia.

Thus, biopsy should be deferred until all auxiliary tests are completed, and all information from medical history and medications used is integrated, providing a full perspective for the decisions regarding the necessity for a biopsy for diagnosis. Judging from the present series, it is suggested that in most of the cases biopsy may not have been truly indicated. One explanation for this relatively high percent of biopsies which were not consistent with SS is the fact that the study was retrospective, spanning many years, during which criteria have been changed, as well as the availability of auxiliary tests has increased. It was also evident that when the referral for biopsy was from a specialist in the oral-dental field, a higher percentage of cases were confirmed SS than in referrals from medical physicians, (50% vs. 19%), indicating a better integration of the relevant clinical parameters prior to decision to perform a lip biopsy. Another factor to be considered is the fact that 3–5% of patients undergoing lip biopsy from minor salivary glands may have complications, such as temporary or persistent sensory deficiency in the area of the biopsy [18]. The surgeon performing the biopsy procedure is aware of these complications and must explain them to the patient in order to obtain informed consent. Although rare, these possible complications should be weighed against the benefit expected, considering the availability of other less invasive methods for diagnosis.

However, the fact that these biopsies accumulated over time in the archives of pathology, provided an opportunity to investigate histopathological findings in non-SS patients, and search for clinical correlations.

A strong correlation between older age and the presence of fatty replacement, fibrosis and acinar atrophy was demonstrated, as well as a strong correlation between fatty replacement and hyperlipidemia. Similar findings regarding age related changes were reported before [19]. However, the correlation of fatty replacement in the salivary glands with hyperlipidemia has not been demonstrated before. The microscopic findings proving replacement of salivary gland parenchyma by fatty tissue provide for the first time a bridge linking the symptoms of xerostomia, the metabolic/biologic process of hyperlipidemia and the pathological changes observed.

Fibrotic changes were found more common in patients without antibodies to SSA and/or SSB, emphasizing the fact that fibrosis is common in patients who have dry mouth due to other causes, and is less prevalent in SS patients.

Although it is well known that elderly patients consume more medications, some of them with recognized xerogenic effects, findings of the current study suggest that anatomic changes in the structure of salivary glands of elderly patients can explain dry mouth symptoms, beyond the pharmacological effect of the medications used.

A rare condition affecting salivary glands, which may mimic SS is IgG4RD. It is a multi-organ immune-mediated fibro-inflammatory condition, with a core of pathological, clinical and serologic similarities [5,6,7,8,9]. The common findings include a tumor-like swelling of involved organs, a lympho-plasmacytic infiltrate enriched in IgG4-positive plasma cells, and a variable degree of fibrosis that has a characteristic storiform pattern. In 60–70% of patients, serum IgG4 is increased [6,7,8,9]. Patients may present with lacrimal and parotid gland enlargement (previously called Mikulicz disease) and/or submandibular gland enlargement (previously called Küttner tumor or sclerosing sialadenitis). These entities were considered in the past to be subcategories of SS but are now considered subcategories of IgG4-related sialadenitis [6,20]. Autoantibodies such as SS-A, SS-B, ANA and RF would be negative, while levels of IgG4 and IgE would be high [8,21,22]. In this present retrospective cohort, no cases consistent with IgG4RD were found, but due to the rarity of this condition, it cannot lead to a clear conclusion regarding involvement of minor salivary glands.

## 5. Conclusions

In conclusion, SS was confirmed in a minority of cases, while xerostomia, in the majority of cases, can be explained by findings of abnormalities in gland structure (acinar atrophy, fatty replacement and fibrosis), and by multiple medications in use. Analysis of medical history and use of available test modalities, such as serology and sialography could lead to a correct diagnosis in non-SS patients, avoiding biopsy. The necessity of a biopsy for diagnosis should be given serious consideration only after all other diagnostic modalities have been employed.

## Figures and Tables

**Table 1:** The correlation between multiple clinical parameters and biopsy results positive or negative for Sjögren syndrome.

Clinical Parameters		Negative Biopsy		Positive Biopsy		*p*-Value
		*N*	%	*N*	%	
Gender	Male	12	28.6	2	16.7	0.71
	Female	30	71.4	10	83.3	
Fibromyalgia	No	38	90.5	12	100	0.564
	Yes	4	9.5	0	0	
Arhtritis	No	40	95.2	12	100	1
	Yes	2	4.8	0	0	
Hypertension	No	32	76.2	9	75	1
	Yes	10	23.8	3	25	
GERD	No	36	85.7	11	91.7	1
	Yes	6	14.3	1	8.3	
Autoimmune disease	No	37	88.1	12	100	0.575
	Yes	5	11.9	0	0	
Hepatitis	No	41	97.6	10	83.3	0.121
	Yes	1	2.4	2	16.7	
Hyperlipidemia	No	30	71.4	9	75	1
	Yes	12	28.6	3	25	
Smoking	No	37	88.1	11	91.7	1
	Yes	5	11.9	1	8.3	
Medications	No	9	22	6	54.5	0.058
	Yes	32	78	5	45.5	
Dry mouth	No	4	9.5	1	8.3	1
	Yes	38	90.5	11	91.7	
Dry eye	No	20	47.6	5	41.7	0.715
	Yes	22	52.4	7	58.3	
Arthralgia	No	38	90.5	9	75	0.175
	Yes	4	9.5	3	25	
Ocular complaints	No	9	23.1	3	25	1
	Yes	30	76.9	9	75	
Schirmer test	Negative	2	66.7	2	100	1
	Positive	1	33.3	0	0	
Topical treatment	Negative	36	87.8	11	91.7	1
	Positive	5	12.2	1	8.3	
Referring physician	Other	25	64.1	6	50	0.502
	Expert *	14	35.9	6	50	
SSA	Negative	31	93.9	5	50	0.004
	Positive	2	6.1	5	50	
SSB	Negative	31	93.9	6	60	0.02
	Positive	2	6.1	4	40	
ANA	Negative	22	64.7	3	37.5	0.235
	Positive	12	35.3	5	62.5	
RF	Negative	17	73.9	5	62.5	0.66
	Positive	6	26.1	3	37.5	

* SSA-Sjogren antibody A, SSB-Sjogren antibody B, ANA-Antinuclear Antibody, RF-Rhematoid factor.

**Table 2:** The correlation between age and microscopic findings of fibrosis, fatty replacement, focal lymphocytic infiltration and acinar atrophy.

		Age						
		N	Mean	Standard Deviation	Median	Minimum	Maximum	*p*-Value
Fibrosis	No	16	50.06	10.52	47.00	36.00	67.00	0.023
	Yes	47	57.57	12.85	59.00	20.00	78.00	
Fatty changes	No	26	51.12	12.10	50.50	27.00	73.00	0.010
	Yes	37	58.86	12.20	60.00	20.00	78.00	
Focal Lymphocytic infiltration	No	16	57.88	11.80	59.50	42.00	76.00	0.520
	Yes	48	55.23	13.02	58.00	20.00	78.00	
Acinar atrophy	No	26	49.50	12.64	47.50	20.00	68.00	0.003
	Yes	37	60.00	10.88	60.00	33.00	78.00	

## Data Availability

Data not published.

## Abbreviations

SS	Sjogren’s Syndrome
IgG4RD	IgG4 related disease
RF	Rheumatoid Factor
ANA	Anti Nuclear Antibody
IgG4	Immunoglobulin G4
GERD	gastro esophageal reflux disease
ST	Schirmer test
